# Supplementary material for: Alcohol reduction outcomes following brief counseling among adults with HIV in Zambia: A sequential mixed methods study
Source: PLOS Glob Public Health. 2022 May 25;2(5):e0000240. doi: 10.1371/journal.pgph.0000240 (PMC10021288; doi:10.1371/journal.pgph.0000240)
Supplement: S1 File — (PDF) [file pgph.0000240.s001.pdf]

FOCUS GROUP DISCUSSION GUIDE

I. INTRODUCTION

My name is __________. We are working with the Centre for Infectious Disease Research in Zambia (CIDRZ) on a project to improve HIV services in Lusaka's communities. We would like to talk to you about your opinions and beliefs surrounding the drinking of alcohol in Lusaka in general and in relation to taking antiretroviral drugs (ARVs) and other HIV care services. Everything you say during this focus group will be kept confidential. The information that you provide will be used to strengthen and improve health services in Zambia.

Remember, you don't have to talk about anything you don't want to and you may end the interview at any time. This discussion will take around one and a half hours. If you have questions you want to ask on other topics, I will help you to find answers after the focus group discussion is over.

*(**NOTE FOR INTERVIEWER: Go through the informed consent for focus group discussions out loud** and give each participant a copy. Ask permission to tape record the discussion, and if they agree, start the tape recorder AFTER the introductions part of the discussion. This guide includes the topics to be covered and questions that may be helpful in facilitating the focus group discussion. You do NOT have to ask all the questions or follow the order given in the guide. Major topic areas and questions are indicated.)*

II. INTRODUCTIONS

First let's get to know each other. Let's go around the circle and each person can introduce himself. You can tell us your first name (or a name you would like to use in this group discussion), your favorite food, and anything else about yourself that you would like to tell the group. (Members of the research team should also introduce themselves. If the group agreed to the tape recording, you may start recording after this section of the discussion.)

III. DISCUSSION TOPICS

Community perceptions, beliefs, and norms surrounding alcohol consumption in urban Zambia in general.

1. How much and how often do people in your community drink alcohol? [men/women; health care workers; community leaders such as pastors, politicians, etc.]
2. What would in your opinion be described as moderate drinking and what would be heavy (too much) drinking? [Note: six or more drinks on one occasion?] How much drinking is dangerous to your health?
3. What type of alcoholic drinks are sold in your community? Which ones are most popular and why? [Note: How is beer different from other alcoholic drinks?]
4. Is drinking different types of alcohol on the same day common? If so, why? What types of alcohol are mixed together? [Note: beer mixed with spirits?]
5. What types of people do you think mostly engage in drinking? [Unemployed/employed, men/women, married/single?] Is it different when women engage in drinking?

Circumstances, patterns, and motivations to drink and to abstain from alcohol consumption.

6. What are some reasons/motives why people in your community engage in drinking alcohol? [social benefits, celebrations, stress relief, pleasure, enjoyment]
7. When do people in your community drink the most: morning, afternoon, after work, weekend? Are people in your community drinking more often in a group or in isolation?
8. Where do most people drink? Outside of taverns/bars are there other places where people drink alcohol? Are there places where drinking is not allowed/not okay?
9. When is drinking not accepted?
10. What are some social situations when people are drinking most? What do people mostly answer when they are offered a drink? Is a person expected to drink? What do people think about a person who refuses to drink or a person who doesn't drink at all? What do people think about a person who drinks "too much"?
11. How do finances affect how much a person drinks alcohol?
12. How does drinking affect work/employment?
13. What do people in the community believe about the negative effects of drinking?

Drinking in urban Zambia in relation to HIV diagnosis, ART, and other HIV care services.

14. How does drinking influence the decision to go for HIV testing?
15. How does drinking influence the decision to initiate ART treatment?
16. When first diagnosed with HIV, is drinking generally discussed with patients? How do the people in the health facility counsel patients regarding drinking alcohol?
17. Do newly diagnosed people with HIV usually truthfully disclose how much and how often they are drinking to their health care providers? Why or why not?
18. What are the effects of drinking alcohol on how well the ARVs work? Does the type (beer, liquor) and quantity matter?
19. What kind of things can help people who are drinking to come to the clinic for their regular HIV clinic visits? What special challenges do people engaging in drinking face in coming to the clinic for HIV clinic visits?
20. Who in the community can help people reduce/stop drinking?

Other comments

What other comments or suggestions do you have when it comes to alcohol and HIV/ARVs?

IV. CLOSING: Thank you very much for your time. Your responses will be helpful for improving the health of people in your community.

FOCUS GROUP PARTICIPANT CHARACTERISTICS FORM

PLACE:
MODERATOR:
NOTE TAKER:
DATE:
Beginning time: Ending time:

TYPE OF GROUP:
CHARACTERISTICS OF PARTICIPANTS

	1	2	3	4	5	6	7	8
Age in years								
Sex (M/F)								
Highest school grade completed (0-12)								
Marital status (never married, married or cohabitating, divorced or separated, widowed)								
