# Supplementary material for: Alcohol reduction outcomes following brief counseling among adults with HIV in Zambia: A sequential mixed methods study
Source: PLOS Glob Public Health. 2022 May 25;2(5):e0000240. doi: 10.1371/journal.pgph.0000240 (PMC10021288; doi:10.1371/journal.pgph.0000240)
Supplement: S2 File — (PDF) [file pgph.0000240.s002.pdf]

IN-DEPTH INTERVIEW GUIDE

I. ICANTASHI

Ishina lyandi nine ___________________________. Tulebombela pamo na ba Centre for Infectious Disease Research in Zambia (CIDRZ) pali pulojekiti iyakuwamya bwino ubwafwilisho ubwa HIV mu makomyuniti aya mu Lusaka. Kuti twatemwa ukulanda naimwe palwa matontonkanyo yenu elyo nefisumino ifyashinguluka iminwine iya ifikola mu Lusaka elyo nokukuma ku kunwa umuti uwa antiretroviral drugs (ARVs) elyo nobwafwilisho bumbi ubwa kutangata pali HIV. Fyonse ifyo mwalalanda panshita iya uku ukulanshanya ukwa mepusho fyalasungwa mu nkama. Ilyashi ilyo mwalapeela likabomfeshiwa mukukosha elyo nokuwamya bwino ubwafwilisho ubwa bumi mu Zambia.

Ibukisheni, tamufwile ukulanda pa cili conse ico tamulefwaya ukulandapo elyo limbi kuti mwaputwisha ukulanshanya ukwa mepusho pa nshita iliyonse. Uku ukulanshanya ukwa mepusho kwalasenda mupepi ne nsa imo na hafu. Nga cakutila namukwata amepusho ayo mulefwaya ukwipusha pa tumitwe tumbi utwelyashi, kuti namwafwilisha ukusanga amasuko panuma iyakupwa kwa kulanshanya ukwa mepusho.

*(**NOTE FOR INTERVIEWER:** **Go through the informed consent form for in-depth interview participants out loud** and give the participant a copy. If he/she agrees to participate, ask him/her to sign the informed consent form. Complete the participant characteristics form. Ask permission to tape record the discussion, and if he/she agrees, start the tape recorder. If he/she refuses, take detailed notes. Complete the introductions part of the discussion. This guide includes the topics to be covered and questions that may be helpful in facilitating the interview. You do NOT have to ask all the questions or follow the order given in the guide.)*

II. UTUMITWE UTWELYASHI UTWAKULANSHANYA

Ificitika ku muntu palwakwe, imisango, elyo nefilengesha ukunwa elyo nokuleka ukunwa sana.

1. Bushe mutontonkanya shani palwa kunwa ifikola?
2. Bushe inshita shimo mulasangwa/mwalesangwa namunwa? Bushe kuti mwanjebako ifyafulilapo palwa minwine yenu? (Probe pali nililali, ni kwisa, bwingi shani, miku inga, musango nshi uwa ifikola)
3. Nga cakutila tamunwa ifikola, bushe kuti mwanjebako palwa milandu isho tamunwina ifikola?
4. Bushe kuti mwanjebako palwa umuntu uli onse uwa mupepi na imwe uyo uunwa ifikola? Bushe ici cimukuma shani?
5. Nga cakutila mwalenwa nangu pali ino inshita mulanwa ifikola, bushe cinshi caba/icali cimo ica mulandu/ icilengesha imwe ukusangwa mulenwa? [ukunonkelamo ukwa kwampana pamo, ukusefya, ukupwisha amasakamiko, ukwanga, ukusekelela]
6. Londololeni Ificitika fimo mukwampana pamo lintu munwa/mwalenwa sana?
7. Bushe musango nshi uwa ifikola ifyo mwatemwapo/mwatemenwepo?

8. Bushe ukunwa ifikola kwakuma shani ubumi ubwa palwenu, ukufikilisha ififwaikwa mu mikalile, ubuntunse bwenu ubwa mu mikalile mu komyuniti, ukucita imilimo yenu iya pa ncito nangu iya pa ng'anda?
9. Bushe mulaitontonkanya mwebene ukuti "mulanwa sana"? Bushe ico cipilibula finshi kuli imwe?
10. Bushe ukunwa ifikola kwakuma shani ku bumi bwenu?
11. Bushe bambi abantu batontonkanya shani palwa minwine yenu?

Ifyo mwapitamo palwenu mu minwine ukukuma ku kusangwa na HIV, ART, elyo nobwafwilisho bumbi ubwa kutangata pali HIV.

12. Bushe mukwai kuti mwalondolola ifyo mwapitamo ilyo mwasangilwe na HIV?
13. Bushe ukunwa kukuma shani ukukwanisha kwenu ukucita na HIV?
14. Lintu mwalembeshiwe ku kiliniki iya ART umuku uwakubalilapo, bushe abapeela ukutangata pa bumi balilanshenyepo na imwe palwa kunwa elyo nga nifyo, bushe musango nshi uwa fintu ifyo bamwipwishepo nangu ukumwebapo? Nga cakutila mwalibebele palwa minwine yenu, bushe bacitile shani?
15. Mu matontonkanyo yenu, bushe abalwele pa kiliniki iya ART mucine-cine balasokolola ubwingi elyo ne miku iyo banwa ifikola ku babomfi? Mulandu nshi nangu mulandu nshi teifyo?
16. Bushe ukunwa ifikola kukuma shani ku kunwa kwenu umuti uwalembwa? Bushe ama ARVs yenu kuti yanwikwa pamo nefyakunwa ifikola?
17. Ukufuma apo mwasangilwe na HIV, bushe mwalicinja imisango iyaminwine iya ifikola? Nga nifyo, bushe kwacinja shani elyo mulandu nshi? [nalicefyako, nalifushako, nalilekelela]
18. Ukufuma apo mwatampile ukunwa ama ARVs, bushe mwalicinja imisango iyaminwine iya ifikola? Nga nifyo, bushe kwacinja shani elyo mulandu nshi? [nalicefyako, nalifushako, nalilekelela]
19. Nga cakutila nacilinga, bushe mulandu nshi mwacefyanyako/ukuleka ukunwa? [amalangililo ayakuleka ukunwa ukufuma ku babomfi abatangata pa bumi, ukufulunganya ukukwanisha ifyakucita mu mikalile iyakwampana pamo nabanandi, icintinya icabucingo ubwa lupiya, icintinya ica mu mikalile iyakwampana pamo nabanandi mu komyuniti].
20. Nga cakutila nacilinga, bushe mwakwanisha shani ukucefyanyako iminwine iya ifikola?
21. Nga cakutila mwalefwaya, bushe ni nani mu komyuniti engamwafwilisha ukucefyanyako/ukuleka ukunwa? Bushe mulaibimba mwibumba iliyonse iyakutungilila mu komyuniti yenu?
22. Bushe mafya nshi ayaibela ayo abantu abaibimba mu mukunwa bakwata ukulaisa ku kiliniki ku butandashi bwabo ubwa ku kiliniki kukutangatwa HIV yabo?
23. Nga cakutila amabumba ayaibela ayakutungilila nangu kanseling'i iya muntu umo na umo yalipangilwe ku balwele abo abanwa, bushe nikwisa uko ifi fifwile ukucitikila elyo mulandu nshi? [mu komyuniti, pa ncende iya bumi, mu macalici]

Fimbi Ifyakukomailapo
Bushe finshi fimbi ifyakukomailapo nangu amatontonkanyo ayo mukwete ukulanda palwa ifikola elyo na HIV/ARVs?

III. UKWISALA
Namitotela sana pa nshita yenu. Amasuko yenu yakafwilisha sana mukuwamya bwino ubumi ubwa bantu mu komyuniti yenu.

(Correct any important misconceptions and provide referrals to counselling and ARV services, if appropriate.)
