# Supplementary material for: Alcohol reduction outcomes following brief counseling among adults with HIV in Zambia: A sequential mixed methods study
Source: PLOS Glob Public Health. 2022 May 25;2(5):e0000240. doi: 10.1371/journal.pgph.0000240 (PMC10021288; doi:10.1371/journal.pgph.0000240)
Supplement: S3 File — (PDF) [file pgph.0000240.s003.pdf]

FOCUS GROUP DISCUSSION GUIDE

I. CHOYAMBA

Dzina langa ndine______________________. Tikusewenzera pamodzi ndi a Centre for Infectious Disease Research in Zambia (CIDRZ) pa pulojekiti ya kukonza bwino mathandizo ya HIV mu ma komyuniti ya mu Lusaka. Tingakonde kukamba ndi inu pa za maganizo anu ndi zikhuluphiliro zozungulira pa nkhani ya kamwedwe ka zolezeretsa mu Lusaka ndipo zokhuzana ndi kumwa mankhwala ya ma antiretroviral (ARVs) ndi mathandizo ena a chisamaliro cha HIV. Zonse zimene muzakamba pa nthawi ya kukambisana kwa mugulu zizakhala za chisinsi. Nkhani zimene muzapasa zizasewenzesedwa kulimbisa ndi kukonza bwino mathandizo a za umoyo mu Zambia.

Kumbukilani, simufunika kukamba pa chilli chonse chimene simufuna kukamba ndipo mwina mungajubizile kukambisana kwa mafunso pa nthawi ili yonse. Uku kukambisana kuzatenga pafupi-fupi ola imodzi ndi hafu. Ngati muli ndi mafunso amene mufuna kufunsa pa tumitu twina twa nkhani, nizakuthandizani kupeza mayankho pambuyo pakuti kukambisana kwa mugulu kwasila.

*(**NOTE FOR INTERVIEWER: Go through the informed consent for focus group discussions out loud** and give each participant a copy. Ask permission to tape record the discussion, and if they agree, start the tape recorder AFTER the introductions part of the discussion. This guide includes the topics to be covered and questions that may be helpful in facilitating the focus group discussion. You do NOT have to ask all the questions or follow the order given in the guide. Major topic areas and questions are indicated.)*

II. CHOYAMBA

Tiyeni tiziwane wina ndi munzake. Tiyeni tiyende mozungulira ndipo munthu aliyense angazikambile yekha kuti nindani. Mungatiuze dzina lanu loyambilila (kapena dzina limene mukufuna kusewenzesa muli uku kukambisana kwa mugulu), chakudya chimene mukonda kwambiri, ndi chibna chili chonse pa za inu nokha chimene mungakonde kuuza gulu. (Members of the research team should also introduce themselves. If the group agreed to the tape recording, you may start recording after this section of the discussion.)

III. TUMITU TWA NKHANI TOKAMBISANAPO

Maganizo, zikhuluphiliro ndi zikhalidwe za mu komyuniti zozungulira kamwedwe ka zolezeretsa mu matauni a mu Zambia monse-monse

1. Kodi ni zolezeretsa zambiri bwanji ndipo ni kangati pamene anthu mu komyuniti yanu amamwa zakumwa zolezeretsa? [azibambo/azimai; anchito za chisamaliro cha umoyo; atsogoleri a mu komyuniti monga azibusa, andale, etc.]
2. Mu maganizo anu, kodi mungafotokoze bwanji kuti uku ndiye kumwa chakumwa cholezeretsa mosapitilira ndipo kumwa chakumwa cholezeretsa mopitilira nikwa bwanji (mowa wambiri)? [Dziwani ichi: zakumwa zolezeretsa zili 6 kapena kupambanapo pa chochitika chimodzi?] Kodi ni kumwa chakumwa cholezeretsa kwa bwanji kwamene kuli koopysa ku umoyo wanu?

3. Kodi ni mutundu bwanji wa zakumwa zolezeretsa zimene zimagulisidwa mu komyuniti yanu? Kodi ni ziti zimene zili zoziwika kwambiri ndipo n’chifukwa chiyani? [Dziwani ichi: Kodi mowa wa mapainti usiyana bwanji ndi zakumwa zina zolezeretsa?]
4. Kodi kumwa zakumwa zolezeretsa za mutundu wosiyana-siyana pa tsiku limodzi kuli kofala? Ngati ni choncho, n’chifukwa chiyani? Kodi ni mitundu bwanji ya zakumwa zolezeretsa zimene zimasankhanisidwa pamodzi? [Dziwani ichi: mowa pamodzi ndi ma sipiliti?]
5. Kodi ni mitundu ya anthu otani amene muganiza kuti kambiri amapezeka akumwa? [Alova/osewenza, azibambo/azimai, Okwatira(wa)/ ankungulume?] Kodi nikosiyana pamene azimai apezeka akumwa?

Zochitika, mutundu, ndi zolengesa kuti anthu azimwa ndi kupewa kumwa zakumwa zolezeretsa

6. Kodi ni zifukwa/ zolengesa zina ziti zimene anthu mu komyuniti yanu amapezeka akumwa zakumwa zolezeretsa? [maphindu ya mu kakhalidwe, zisangalalo, kuchepesa nkhawa, kusangalala, kukondwela]
7. Kodi ni nthawi iti imene anthu mu komyuniti yanu kambiri amamwa?: kuseni, muzuba, pambuyo pa kusewenza, pa mapeto ya m’lungu? Kodi anthu mu komyuniti yanu amamwa mopambana kwambiri mu gulu kapena pa yekha-pa yekha?
8. Kodi ni kuti kwamene kambiri anthu amamwela? Kunja kwa mataveni/ma biyaolo? Kodi kuli malo ena kwamene anthu amamwela zakumwa zolezeretsa? Kodi kuli malo kwamene kumwa zolezeretsa sikololedwa/ sikuli bwino?
9. Kodi ni liti pamene kumwa sikovomerezedwa?
10. Kodi ni pa zochitika zina zotani za chikhalidwe pamene anthu kambiri amapezeka akumwa? Kodi ni chiyani chimene anthu kambiri amayankha pamene amapasidwa chakumwa? Kodi munthu amayembekezeredwa kumwa? Kodi anthu amaganiza chiyani pa za munthu akana kumwa kapena munthu amene samamwa ngakhale pang’ono? Kodi anthu amaganiza chiyani pa za munthu amene amamwa “kwambiri”?
11. Kodi ndalama zimakhuza bwanji mwamene munthu amamwela zakumwa zolezeretsa?
12. Kodi kumwa kumakhuza bwanji kusewenza/nchito?
13. Kodi anthu mu komyuniti amakhulupilira chiyani pa zotulukamo zoipa za kumwa?

Kumwa mu matauni a mu Zambia mokhuzana ndi kupeza HIV, ART, ndi mathandizo ena a chisamaliro cha HIV

14. Kodi kumwa kumalengesa bwanji kuti munthu asankhe kupimisa HIV?
15. Kodi kumwa kumalengesa bwanji kuti munthu asankhe kuyamba kumwa mankhwala ya ART?
16. Nthawi yoyamba pamene munthu apezeka ndi HIV, kodi kumwa kambiri kumakambidwapo pamodzi ndi odwala? Kodi anthu mu malo a za umoyo amawauza bwanji odwala kukhuzana ndi kumwa zakumwa zolezeretsa?
17. Kodi anthu amene apezedwa ndi HIV chasopano kambiri moona mtima amaulula kupaka ndi kambiri kamene amamwa zolezeretsa kwa anchito za chisamaliro cha umoyo wao? N'chifukwa chiyani kapena n'chifukwa chiyani samatero?
18. Kodi ni zotulukamo za bwanji za kumwa zakumwa zolezeretsa pa mwamene ma ARVs amasewenzera bwino? Kodi mutundu (mowa, masipiliti) ndi kupaka kuli ndi kanthu?
19. Kodi ni mutundu uti wa zinthu zimene zingathandize anthu amene akumwa kuti azibwera ku kutandala kwao nthawi zonse kwa ku kiliniki kwa HIV? Kodi ni mavuto yati yapadera yamene anthu amene amapezeka akumwa amakhala nao kubwera ku kiliniki ku kutandala kwa ku kiliniki kwa HIV?
20. Kodi ni ndani mu komyuniti angathandize anthu kuchepesako/kuleka kumwa?

ZOKAMBAPO ZINA
Kodi ni zokambapo zina zotani kapena maganizo anu amene muli nayo pa nkhani ya zakumwa zolezeretsa ndi HIV/ARVs?

IV. KUSILIZA: Zikomo kwambiri pa nthawi yanu. Mayankho anu azakhala othandiza kuti tikakonze bwino umoyo wa anthu mu komyuniti yanu.

FOCUS GROUP PARTICIPANT CHARACTERISTICS FORM

PLACE:
MODERATOR:
NOTE TAKER:
DATE:
Beginning time: Ending time:

TYPE OF GROUP:
CHARACTERISTICS OF PARTICIPANTS

	1	2	3	4	5	6	7	8
Age in years								
Sex (M/F)								
Highest school grade completed (0-12)								
Marital status (never married, married or cohabitating, divorced or separated, widowed)								
