# Supplementary material for: Alcohol reduction outcomes following brief counseling among adults with HIV in Zambia: A sequential mixed methods study
Source: PLOS Glob Public Health. 2022 May 25;2(5):e0000240. doi: 10.1371/journal.pgph.0000240 (PMC10021288; doi:10.1371/journal.pgph.0000240)
Supplement: S4 File — (PDF) [file pgph.0000240.s004.pdf]

IN-DEPTH INTERVIEW GUIDE

I. INTRODUCTION

My name is ________. We are working with the Centre for Infectious Disease Research in Zambia (CIDRZ) on a project to improve HIV services in Lusaka's communities. We would like to talk to you about your opinions and beliefs surrounding drinking of alcohol in Lusaka in general and in relation to taking antiretroviral drugs (ARVs) and other HIV care services. Everything you share during this interview will be kept confidential. The information that you provide will be used to strengthen and improve health services in Zambia.

Remember, you don't have to talk about anything you don't want to and you may end the interview at any time. This interview will take around one and a half hours. If you have questions you want to ask on other topics, I can assist you to find answers after the interview is over.

*(**NOTE FOR INTERVIEWER:** **Go through the informed consent form for in-depth interview participants out loud** and give the participant a copy. If he/she agrees to participate, ask him/her to sign the informed consent form. Complete the participant characteristics form. Ask permission to tape record the discussion, and if he/she agrees, start the tape recorder. If he/she refuses, take detailed notes. Complete the introductions part of the discussion. This guide includes the topics to be covered and questions that may be helpful in facilitating the interview. You do NOT have to ask all the questions or follow the order given in the guide.)*

II. DISCUSSION TOPICS

Personal circumstances, patterns, and motivations to drink and to abstain from heavy drinking in general.

1. What do you generally think about drinking alcohol?
2. Do/did you sometimes engage in drinking? Can you tell me more about your drinking? (Probe on when, where, how much, how often, what types of alcohol)
3. If you do not drink alcohol, can you tell me about the reasons that you don't drink?
4. Can you tell me about anyone close to you who drinks alcohol? How does that affect you?
5. If you used to or current drink alcohol, what are/were some reasons/motives for you to engage in drinking? [social benefits, celebrations, stress relief, pleasure, enjoyment]
6. Describe some social situations when you drink/drank the most?
7. What type of drink do/did you prefer?
8. How has drinking alcohol affected your personal life, social obligations, social standing in the community, performing your responsibilities at work or around the house?
9. Do you consider yourself a "heavy drinker"? What does that mean to you?
10. How has drinking alcohol affected your health?
11. What do other people think about your drinking?

Personal experiences with drinking in relation to HIV diagnosis, ART, and other HIV care services.

12. Can you please describe your experience being diagnosed with HIV?
13. How does drinking influence your ability to deal with HIV?
14. When first enrolled at ART clinic, did health care providers discuss drinking with you and if so, what types of things did they ask you or tell you? If you told them about your drinking, how did they react?
15. In your opinion, do patients at ART clinic truthfully disclose how much and how often they are drinking to the staff? Why or why not?
16. How does drinking alcohol affect your taking of your prescribed medications? Can your ARVs be taken with alcohol?
17. Since you have been diagnosed with HIV, have you changed your drinking patterns? If so, how has it changed and why? [reduced, increased, stopped altogether]
18. Since you started taking ARVs have you changed your drinking patterns? If so, how has it changed and why? [reduced, increased, stopped altogether]
19. If applicable, why did you reduce/abstain from drinking? [advice against drinking from health care workers, interference with fulfillment of social obligations, threats to financial security, threats to social standing in the community].
20. If applicable, how did you manage to reduce your alcohol intake?
21. If you wanted to, who in the community could help you to reduce/stop drinking? Do you engage in any support group in your community?
22. What special challenges do people engaging in drinking face in coming to the clinic for HIV clinic visits?
23. If special support groups or one-to-one counseling were created for patients who drink, where should these take place and why? [in the community, facility, churches]

Other comments

What other comments or suggestions do you have when it comes to alcohol and HIV/ARVs?

III. CLOSING

Thank you very much for your time. Your responses will be very helpful for improving the health of people in your community.

(Correct any important misconceptions and provide referrals to counselling and ARV services, if appropriate.)
