# Supplementary material for: Alcohol reduction outcomes following brief counseling among adults with HIV in Zambia: A sequential mixed methods study
Source: PLOS Glob Public Health. 2022 May 25;2(5):e0000240. doi: 10.1371/journal.pgph.0000240 (PMC10021288; doi:10.1371/journal.pgph.0000240)
Supplement: S5 File — (PDF) [file pgph.0000240.s005.pdf]

FOCUS GROUP DISCUSSION GUIDE

I. ICANTASHI

Ishina lyandi nine ___________________________. Tulebombela pamo na ba Centre for Infectious Disease Research in Zambia (CIDRZ) pali pulojekiti iyakuwamya bwino ubwafwilisho ubwa HIV mu makomyuniti aya mu Lusaka. Kuti twatemwa ukulanda naimwe palwa matontonkanyo yenu elyo nefisumino ifyashinguluka iminwine iya ifikola mu Lusaka elyo nokukuma ku kunwa umuti uwa antiretroviral drugs (ARVs) elyo nobwafwilisho bumbi ubwa kutangata pali HIV. Fyonse ifyo mwalalanda panshita iya uku ukulanshanya ukwa mwibumba fyalasungwa mu nkama. Ilyashi ilyo mwalapeela likabomfeshiwa mukukosha elyo nokuwamya bwino ubwafwilisho ubwa bumi mu Zambia.

Ibukisheni, tamufwile ukulanda pa cili conse ico tamulefwaya ukulandapo elyo limbi kuti mwaputwisha ukulanshanya ukwa mepusho pa nshita iliyonse. Uku ukulanshanya kwalasenda mupepi ne nsa imo na hafu. Nga cakutila namukwata amepusho ayo mulefwaya ukwipusha pa tumitwe tumbi utwelyashi, kuti namwafwilisha ukusanga amasuko panuma iyakupwa kwa kulanshanya ukwa mwibumba.

*(**NOTE FOR INTERVIEWER: Go through the informed consent for focus group discussions out loud** and give each participant a copy. Ask permission to tape record the discussion, and if they agree, start the tape recorder AFTER the introductions part of the discussion. This guide includes the topics to be covered and questions that may be helpful in facilitating the focus group discussion. You do NOT have to ask all the questions or follow the order given in the guide. Major topic areas and questions are indicated.)*

II. INTRODUCTIONS

Icakubalipo lekeni twishibane umo nomunankwe. Lekeni tuleshinguluka elyo cila muntu aleilandila umwine eka ukuti ninani. Kuti mwatweba ishina lyenu ilyakubalilapo (nangu ishina ilyo mwingatemwa ukubomfya muli uku ukulanshanya ukwa mwibumba), icakulya ico mwatemwisha, elyo nacimbi icili conse palwa imwebene ico mwingatemwa ukweba ibumba. (Members of the research team should also introduce themselves. If the group agreed to the tape recording, you may start recording after this section of the discussion.)

III. UTUMITWE UTWELYASHI UTWAKULANSHANYA

Amatontonkanyo aya mu komyuniti, ifisumino, elyo nentambi ishashunguluka iminwine iya ifikola mu matauni aya mu Zambia monse-monse.

1. Bushe bwingi shani elyo miku inga iyo abantu mu komyuniti yenu banwa ifyakunwa ifikola? [bashibantu/banamayo; ababomfi abatangata pa bumi; intungulushi isha mu komyuniti pamo nga bashimapepo, abafikansa fya calo, etc.]
2. Mu matontonkanyo yenu bushe kuti mwalondolola shani ukuti ekunwa ukwalinga elyo ukunwa sana kwa shani? [Ishibeni ici: ifyakunwa ifili 6 nangu ukucilapo pacicitika cimo?] Bushe kunwa sana ukwashani ukwingaleta ubusanso ku bumi bwenu?

3. Bushe musango shani uwa ifyakunwa ifikola ifishitishiwa mu komyuniti yenu? Bushe niyesa ayaseeka sana elyo mulandu nshi? [Ishibeni ici: Bushe ubwalwa bwapusana shani kuli fimbi ifyakunwa ifikola?]
4. Bushe ukunwa ifyakunwa ifikola ifyapusana-pusana pa bushiku bumo bwine kwaliseeka? Nga ni fyo, mulandu nshi? Bushe musango nshi uwa ifyakunwa ifikola ifisankanyishiwa pamo? [Ishibeni ici: ubwalwa ukusankanya na ma sipiliti?]
5. Bushe musango nshi uwa bantu abo mutontonkanya ukuti ilingi line ebasangwa mukunwa? [Amalofwa/ababomba, bashibantu/banamayo, abaupwa/bankungulume?] Bushe cilaba icapusanako lintu banamayo balesangwa mukunwa?

Ifificika, imisango, elyo nefilengesha abantu balenwa elyo nokuleka ukunwa ifyakunwa ifikola.

6. Bushe milandu shimo/ifilengesha isho abantu mu komyuniti yenu basangilwa balenwa ifyakunwa ifikola? [ukunonkelamo ukwa kwampana pamo, ukusefya, ukupwisha amasakamiko, ukwanga, ukusekelela]
7. Bushe nililali lintu abantu mu komyuniti yenu banwa ilingi line: ulucelo, akasuba, panuma iya milimo, pa mpela ya mulungu? Bushe abantu mu komyuniti yenu imiku iyingi balanwa mwibumba nangu nangu beka-beka?
8. Bushe ni kwisa ujko abantu abengi banwina? Kunse iya mataveni/biyaholo? Buse kwalibako incende shimbi uko abantu banwina ifyakunwa ifikola? Buse kwalibako incende shimbi uko ukunwa takwasuminishiwa/ takwaba bwino?
9. Bushe nililali lintu ukunwa takwasuminishiwa?
10. Bushe fyakucitika nshi fimo ifyakwampana pamo nabanenu lintu abantu abanwa ilingi line? Bushe abantu ilingi line basuka shani lintu bapeelwa icakunwa? Bushe umuntu alesubilwa ukunwa? Bushe abantu batontonkanya shani palwa muntu uukana ukunwa nangu umuntu uushinwa nangula panono? Bushe abantu batontonkanya shani palwa muntu “uunwa sana”?
11. Bushe ulupiya lukuma shani pafyo umuntu anwa sana ifyakunwa ifikola?
12. Bushe iminwine ikuma shani umulimo/incito?
13. Bushe abantu mu komyuniti basuminamo shani palwa ifitumbukamo ifibi ifya kunwa?

Ukunwa mu matauni aya mu Zambia ukwakuma ku kusanga HIV, ART, elyo nobwafwilisho bumbi ubwakutangata HIV.

14. Bushe iminwine ikuma shani palwa kusala ukuyapimisha HIV?
15. Bushe iminwine ikuma shani palwa kusala ukutampa umuti uwa ART?
16. Pa nshita iyakubalilapo ukusangwa na HIV, bushe ukunwa ilingi line kwalilanshiwepo pamo nabalwele? Bushe abantu pa ncende iya bumi basambilisha shani abalwele ukukuma ku kunwa ifyakunwa ifikola?
17. Bushe abantu basangwa na HIV ica nombaline ilingi line mu cine-cine balasokolola ubwingi elyo ne miku iyo banwa ku babomfi abapeela ukutangata ukwa bumi bwabo? Mulandu nshi nangu mulandu teifyo?
18. Bushe finshi Ifumbukamo mukunwa ifyakunwa ifikola pafyo umuti uwa ma ARVs yabomba bwino? Bushe umusango (ubwalwa, ama sipiliti) elyo nobwingi fyaliba nakantu?
19. Bushe musango nshi uwa fintu uwingafwilisha abantu abanwa ukuti baleisa ku kiliniki ku kutandala kwabo ukwa nshita shonse ukwa ku kiliniki ukwa pali HIV? Bushe mafya nshi ayaibela ayo abantu abaibimba mukunwa bakwata mukwisa ku kiliniki ku kutandala kwabo ukwa ku kiliniki ukwa pali HIV?
20. Bushe nibani mu komyuniti abengafwilisha abantu ukucefyako/ukuleka ukunwa?

Fimbi Ifyakukomailapo
Bushe finshi fimbi ifyakukomailapo nangu amatontonkanyo ayo mukwete ukulanda palwa ifikola elyo na HIV/ARVs?

IV. UKWISALA: Namitotela sana pa nshita yenu. Amasuko yenu yakafwilisha sana mukuwamya bwino ubumi ubwa bantu mu komyuniti yenu.

FOCUS GROUP PARTICIPANT CHARACTERISTICS FORM

PLACE:
MODERATOR:
NOTE TAKER:
DATE:
Beginning time: Ending time:

TYPE OF GROUP:
CHARACTERISTICS OF PARTICIPANTS

	1	2	3	4	5	6	7	8
Age in years								
Sex (M/F)								
Highest school grade completed (0-12)								
Marital status (never married, married or cohabitating, divorced or separated, widowed)								
