# Supplementary material for: Alcohol reduction outcomes following brief counseling among adults with HIV in Zambia: A sequential mixed methods study
Source: PLOS Glob Public Health. 2022 May 25;2(5):e0000240. doi: 10.1371/journal.pgph.0000240 (PMC10021288; doi:10.1371/journal.pgph.0000240)
Supplement: S6 File — (PDF) [file pgph.0000240.s006.pdf]

IN-DEPTH INTERVIEW GUIDE

I. CHOYAMBA

Dzina langa ndine_______________________. Tikusewenzela pamodzi ndi a Centre for Infectious Disease Research in Zambia (CIDRZ) pa pulojekiti ya kukonza bwino mathandizo ya HIV mu ma komyuniti ya mu Lusaka. Tingakonde kukamba ndi inu pa za maganizo anu ndi zikhuluphiliro zozungulira pa nkhani ya kamwedwe ka zolezeretsa mu Lusaka ndipo zokhuzana ndi kumwa mankhwala ya ma antiretroviral (ARVs) ndi mathandizo ena a chisamaliro cha HIV. Zonse zimene muzakamba pa nthawi ya kukambisana kwa mafunso zizakhala za chisinsi. Nkhani zimene muzapasa zizasewenzesedwa kulimbisa ndi kukonza bwino mathandizo a za umoyo mu Zambia.

Kumbukilani, simufunika kukamba pa chilli chonse chimene simufuna kukamba ndipo mwina mungajubizile kukambisana kwa mafunso pa nthawi ili yonse. Uku kukambisana kuzatenga pafupi-fupi ola imodzi ndi hafu. Ngati muli ndi mafunso amene mufuna kufunsa pa tumitu twina twa nkhani, nizakuthandizani kupeza mayankho pambuyo pakuti kukambisana kwa mafunso kwasila.

*(**NOTE FOR INTERVIEWER:** **Go through the informed consent form for in-depth interview participants out loud** and give the participant a copy. If he/she agrees to participate, ask him/her to sign the informed consent form. Complete the participant characteristics form. Ask permission to tape record the discussion, and if he/she agrees, start the tape recorder. If he/she refuses, take detailed notes. Complete the introductions part of the discussion. This guide includes the topics to be covered and questions that may be helpful in facilitating the interview. You do NOT have to ask all the questions or follow the order given in the guide.)*

II. TUMITU TWA NKHANI TOKAMBISANAPO

Zochitika za munthu payekha, mutundu, zolengesa kumwa ndi kupewa kumwa kwambiri monse-monse.

1. Kodi muganiza bwanji kambiri pa za kumwa zolezeretsa?
2. Kodi nthawi zina muma/munapezeka mukumwa? Kodi munganiuzeko zoonjezereka pa zakumwa kwanu? (Probe ni liti, ni kuti, ni wambiri bwanji, ni nthawi zambiri bwanji, ni mutundu uti wa zolezeretsa)
3. Ngati simumamwa zolezeretsa, kodi munganiuzeko pa zifukwa zimene simumwela?
4. Kodi munganiuzeko pa za munthu aliyense wa pafupi kwa inu amene amamwa zolezeretsa? Kodi chimakukhuzani bwanji?
5. Ngati munali kumwa kapena panthawi zino mumamwa zolezeretsa, kodi ni ziti zimene zili/zinali zifukwa/zolengesa zina kuti inu muzipezeka mukumwa? [maphindu ya mu kakhalidwe, zisangalalo, kuchepesa nkhawa, kusangalala, kukondwela]
6. Fotokozani zochitika zina za mu kakhalidwe pamene mumamwa/munamwa kwambiri?
7. Kodi ni mutundu uti wa zolezeretsa wamene munasankhapo?

8. Kodi kumwa zolezeretsa kwakhuza bwanji umoyo wanu, zochita zanu mu chikhalidwe, kaimidwe kanu mu chikhalidwe cha mu komyuniti, kuchita maudindo anu ya pa nchito kapena ya pa nyumba?
9. Kodi mu maziganizila mweka kukhala munthu "wakumwa kwambiri"? Kodi icho chimatanthauza chiyani kwa inu?
10. Kodi kumwa zolezeretsa kwakhuza bwanji umoyo wanu?
11. Kodi anthu ena amaganiza chiyani pa kumwa kwanu zolezeretsa?

Zimene mwapitamo pamweka mukumwa zolezeretsa mokhuzana ndi kupeza HIV, ART, ndi mathandizo ena a chisamaliro cha HIV.

12. Chonde mungafotokoze zimene mwapitamo mwakupezedwa ndi HIV?
13. Kodi kumwa kumakhuza bwanji kukwanisa kwanu kuchita ndi vuto ya HIV?
14. Pamene munalembesedwa ku kiliniki ya ART poyamba, kodi anchito za chisamaliro cha za umoyo anakambisilana ndi inu pa za kumwa ndipo ngati anatero, kodi ni zinthu za mutundu wotani zimene anakufunsani kapena kukuuzani? Ngati munawauza pa za kamwedwe kanu, kodi anachita bwanji?
15. Mukuganiza kwanu, kodi odwala ku kiliniki ya ART moona mtima amaulula kupaka ndi kuti ni kangati kamene amamwa kwa anchito za mu kiliniki? N'chifukwa chiyani kapena n'chifukwa samatero?
16. Kodi kumwa zolezeretsa kumakhuza bwanji kamwedwe kanu ka mankhwala yolembedwa? Kodi ma ARVs yanu yangamwedwe pamodzi ndi mowa?
17. Kuchokera pamene anakupezani ndi HIV, kodi munasintha kamwedwe kanu? Ngati nitero, kodi kwasintha bwanji ndipo n'chifukwa chiyani? [anabwerelako pansi, anaonjezelako, analekelathu]
18. Kuchokera pamene munayamba kumwa ma ART, kodi munasintha kamwedwe kanu? Ngati nitero, kodi kwasintha bwanji ndipo n'chifukwa chiyani? [anabwerelako pansi, anaonjezelako, analekelathu]
19. Ngati ni choncho, kodi n'chifukwa chiyani munachepesako/munaleka kumwa? [malangizo yoleka kumwa kuchokera kwa anchito za chisamaliro cha umoyo, kusokoneza pa nkhani za kukwanirisa zochita za mu chikhalidwe, kuopyezedwa ku chitetezo cha ndalama, kuopyezedwa kaimidwe ka mu chikhalidwe mu komyuniti].
20. Ngati ni choncho, kodi munakwanisa bwanji kuchepesako kamwedwe kanu ka zolezeretsa?
21. Ngati munali kufuna, kodi ndani mu komyuniti angakuthandizeni kuti muchepeseko/ muleke kumwa? Kodi mumapezeka mu gulu ili yonse yothandiza mu komyuniti yanu?
22. Kodi ni mavuto yati yapadera yamene anthu amene amamwa amapitamo mwa kubwera ku kutandala kwa ku kiliniki kwa HIV?
23. Ngati thandizo ya mu magulu yapadera kapena kanseling'i ya munthu umodzi ndi umodzi yanapangidwa kwa odwala amene amamwa, kodi ni kuti kwamene izi zingachitikile ndipo n'chifukwa chiyani? [mu komyuniti, malo a za umoyo, ku machalichi]

ZOKAMBAPO ZINA
Kodi ni zokambapo zina zotani kapena maganizo anu amene muli nayo pa nkhani ya zakumwa zolezeretsa ndi HIV/ARVs?

III. KUSILIZA
Zikomo kwambiri pa nthawi yanu. Mayankho anu azakhala othandiza kuti tikakonze bwino umoyo wa anthu mu komyuniti yanu.

(Correct any important misconceptions and provide referrals to counselling and ARV services, if appropriate.)
