# Supplementary material for: Alcohol reduction outcomes following brief counseling among adults with HIV in Zambia: A sequential mixed methods study
Source: PLOS Glob Public Health. 2022 May 25;2(5):e0000240. doi: 10.1371/journal.pgph.0000240 (PMC10021288; doi:10.1371/journal.pgph.0000240)
Supplement: Attachment — Submitted filename: Response to Reviewers.docx [file pgph.0000240.s008.docx]

**REBUTTAL LETTER**

**EDITOR COMMENT:**

1. Please include a copy of the interview guide used in the study, in both the original language and English, as Supporting Information, or include a citation if it has been published previously.

**RESPONSE:**

**We have included a copy of interview guides used in English and two local languages (Bemba and Nyanja). Of note, lay and professional health worker focus groups were held in English only (no local language guides). These guides have not been published.**

**EDITOR COMMENT:**

2. Please update the completed 'Competing Interests' statement, including any COIs declared by your co-authors. If you have no competing interests to declare, please state "The authors have declared that no competing interests exist". Otherwise please declare all competing interests beginning with the statement "I have read the journal's policy and the authors of this manuscript have the following competing interests:"

**RESPONSE:**

**All authors have no competing interested and this has been documented in the revised manuscript.**

**EDITOR COMMENT:**

3. Please remove the figures embedded in your manuscript file leaving only the separate figures files.

**RESPONSE:**

**We removed the figures and have attached them as separate files.**

**EDITOR COMMENT:**

4. Since your data is not available for proprietary reasons, please explain via email why the data is not available. Please also include the contact information for the third party organization that should be contacted should other researchers want to request access to this data and please include the full citation of where the data can be found. We also request that you verify with us via email that any researcher will be able to obtain the data set in the same manner that the you have obtained it. If you feel you are unwilling or unable to adhere to this policy, please explain your reasons by return email and your exemption request will be escalated to the editor for approval. Your exemption request will be handled independently and will not hold up the peer review process, but will need to be resolved should your manuscript be accepted for publication. One of the Editorial team will be in touch if they require more information.

**RESPONSE:**

**The data are available for use by other researchers but we are unable to upload the data to an open data archive because of Zambian regulatory requirements. If a researcher wants to work with the data, procedures are in place to allow this. First they would contact Dr. Samuel Bosomprah at CIDRZ data center (**[**Samuel.bosomprah@cidrz.org**](mailto:Samuel.bosomprah@cidrz.org)**) or the corresponding author. Then, they would be given a data access form to complete. Thereafter the data can be shared. We can verify that any researcher would be provided with the exact data (both quantitative and qualitative) used in the analysis presented in this paper.**

**EDITOR COMMENT:**

5. Please amend your detailed Financial Disclosure statement. This is published with the article, therefore should be completed in full sentences and contain the exact wording you wish to be

i) State the initials, alongside each funding source, of each author to receive each grant.

iii). State what role the funders took in the study. If the funders had no role in your study, please state: “The funders had no role in study design, data collection and analysis, decision to publish, or preparation of the manuscript.”

**RESPONSE:**

**We have amended the financial disclosure as requested to read as follows:**

**“Research reported in this publication was supported by the National Institutes of Health under Award Number U01AI069924, which is co-funded by National Institute of Child Health and Human Development (NICHD), National Cancer Institute (NCI), National Institute of Mental Health (NIMH), National Institute of Diabetes and Digestive and Kidney Diseases (NIDDK), National Institute on Alcohol Abuse and Alcoholism (NIAAA), National Heart, Lung, and Blood Institute (NHLBI), Fogarty International Center (FIC), National Institute on Drug Abuse (NIDA), National Heart, Lung, and Blood Institute (NHLBI), and the National Institute of Allergy and Infectious Diseases (NIAID).  Additional support for this work came from NIAAA to M.J.V. and K.C. (P01AA029540) and to J.K. (K01AA026523), from NIAID to M.J.V. (P30AI027767), and from the UAB Sparkman Center to A.H., J.T. and M.J.V.**

**In the revised paper, we also state that, “The funders had no role in study design, data collection and analysis, decision to publish, or preparation of the manuscript.”**

**REVIEWER #1 COMMENT:**

This is an informative and relevant study and the findings can potentially have important uses. Please see my comments below. Was the 3-question tool used or the 10-question Audit C tool used?

**RESPONSE:**

**We used the 3-question the Alcohol Use Disorders Identification Test-Consumption (AUDIT-C) tool in this study. This was stated more clearly in the revised manuscript (page 4, paragraph 2) where we have written, “*Alcohol use was measured during enrollment while the patient was being initiated on ART, and at 1 year later using the 3-question Alcohol Use Disorders Identification Test-Consumption (AUDIT-C)”***

**REVIEWER #1 COMMENT:**

Page 7, quantitative methods: What is the variable ‘clinic’ used in regression?

**RESPONSE:**

**Clinic is the clinic name. As described in methods the study occurred at 2 clinics. In the revised paper (page 4, paragraph 2) we wrote, “ The The enrollment clinic (both were large urban sites with 5,000-10,000 PLWHA in care) was noted in each patient’s record to allow for analysis of clinic-level differences .“**

**REVIEWER #1 COMMENT:**

Page 8, qualitative methods: The IDIs were conducted with individuals who self-reported reduced use and persistence use: self-reports are often not very reliable, especially in alcohol users; why did the investigators not objectively assess change in alcohol use over one-year period, since these individuals were being followed-up as part of the study?

**RESPONSE:**

**This is an excellent point and we have acknowledged that self-reported data-the standard in alcohol research-can be biased. We used the AUDIT-C survey at baseline and 1 year and based on the change (from unhealthy to moderate use/abstinence or unhealth use at both time points), we recruited participants for IDIs. We have revised the methods to make this more clear. In the revised paper (page 5, paragraph 1), we stated, “ To explore their perspectives, beliefs, and norms surrounding alcohol consumption in relation to HIV infection and care received at the clinic we facilitated two focus group discussions (FG) with people living with HIV and reporting unhealthy alcohol use (one with men and one with women). To understanding perspectives around alcohol reduction in these individuals, we also conducted twenty in-depth interviews (IDIs) with participants based on their patterns of alcohol use from the AUDIT-C.”**

**REVIEWER #1 COMMENT:**

Page 8, qualitative methods: Anderson`s Behaviour Model (ABM) is supposed to be used as a framework to design the methods as well as interpret the results. While the authors have interpreted the results using ABM, please describe how the model was used as a framework in designing the methods.

**RESPONSE:**

**The study was planned as a cohort study without any qualitative component.**

**After we recognized a high prevalence of persistent unhealthy alcohol use, we adopted the ABM to design a mixed methods analysis of the problem (why cohort participants are continuing to report high levels of alcohol use). Thus, ABM was used in designing the methods, in formulating the interview and focus group guides, and in interpretation of results. We have added some content to the revised paper to give more details.**

**“Our approach in study design, content of interview/focus group guides, and interpretation of data was guided by the adapted Andersen’s Behavioral Model (17), which suggests that determinants of health behaviors include the healthcare environment, patient characteristics, the community, the societal context and patient’s need for health services (17)”.**

**REVIEWER #1 COMMENT:**

Page 8, qualitative methods: Please give more details about the thematic analysis approach.

**RESPONSE:**

**We have included more details about the thematic analysis as follows (see page 5, paragraph 3): “Coding and analysis followed a reflexive thematic analysis approach, in which broad coding framework precedes theme development. The broad coding framework was based on the literature, constructs of the Andersen’s Behavioral Model, topics from interview guide, and in the later phase of coding, based on emerging themes inductively derived from transcripts. Transcripts were initially broad coded by three individuals trained in qualitative coding, and consistency of coding was established. Excerpts from broad codes were then fine coded using an inductive approach.”**

**REVIEWER #1 COMMENT:**

Page 8, quantitative results: Was the sample size statistically calculated? If yes, please give details. If no, please justify the sample size.

***RESPONSE:***

**The overall cohort sample size, designed in 2011, was initially based on this being a 3-site study (Zambia, Mozambique, and South Africa) and on the need to compare people with HIV-HBV coinfection and HIV alone. We expected that 3-6% would be hepatitis B surface antigen positive, and we wanted to compare people with HiV-HBV coinfection to those with HIV alone with sufficient power to detect a hazard ration of 1.7. Thus, a target of 3000 (1,000 per country ) was proposed. Unfortunately, in 2012-3, Mozambique and South African sites had significant challenges implementing the study and resourcecs were redirected to focus on Zambia. After the initial 797 were enrolled in Zambia (and 97 had HIV-HBV; 12.1%), to have more power to compare people with HIV-HBV and HIV only, we narrowed eligibility to those with HIV-HBV coinfection. WE ultimately recruited 897 participants, including 197 with HIV-HBV. This yielded use reasonable power for the main analyses.**

**Alcohol use was not factored into the sample size calculation as this was one of the first times we consistently and longitudinally measured it.**

**REVIEWER #1 COMMENT:**

Page 8, quantitative results: Consecutive enrolment was used as reported by the authors; this raises the question of how representative the study population was of the general population. In such a method, it is likely that heavy drinkers or PLWHs with severe illness may not participate in the study and thus an important stratum of alcoholic PLWHs may have been left out of the study. This should be mentioned as a study limitation.

***RESPONSE:***

**Enrollmenet occurred during the entire clinic session and hence those who came later because of illness or alcohol use with a hangover would have potentially joined. In general, very sick patients were not enrolled as they needed to be stable to sign consent and we needed to believe they could do a longitudinal cohort of this kind. We agree with the reviewer that our enrollment approach would have excluded some patients and thus slightly reduced the representativeness of the data.**

**REVIEWER #1 COMMENT:**

Page 8, quantitative results: The Audit C tool scores are interpreted as low, increasing and high risk and dependence. There are not reported in the manuscript. Further, what is the definition of unhealthy alcohol use and moderate alcohol use and how have the authors calculated these? The authors should maintain consistency between methods and results.

**RESPONSE:**

**We used the 3-question the Alcohol Use Disorders Identification Test-Consumption (AUDIT-C) tool in this study. This was stated more clearly in the revised manuscript (page 4, paragraph 2) where we have written, “*Alcohol use was measured during enrollment while the patient was being initiated on ART, and at 1 year later using the 3-question Alcohol Use Disorders Identification Test-Consumption (AUDIT-C)”***

**REVIEWER #1 COMMENT:**

Page 9, quantitative results: Regression analysis should adjust for some measure of limited alcohol-reduction counselling, i.e., whether given or not, how many times, etc., since this variability in this intervention can potentially alter alcohol consumption practices on its own. Also, the authors state that one of the conclusions from this study is that current counselling practices are inadequate. However, without knowing about the details of this counselling and accounting for this counselling in analysis, such a conclusion cannot be made. While the authors have mentioned this as a limitation, is it possible to go back, search for data regarding this variable and redo the regression analysis? If this is not possible, the authors should remove this sentence from conclusion, discussion and abstract sections.

**RESPONSE:**

**We agree with the reviewers sentiments here. Unfortunately, this cohort was not established to assess alcohol use outcomes and/or the strengths and limitation of counseling. There is no way to go back and add that specific data. We agree that we cannot comment on the quality of current counseling practices and we have removed any mention that the current counseling approaches are inadequate. All we can say is that receipt of standard of HIV care led to a moderate reduction but some had persistent unhealthy alcohol use.**

**REVIEWER #1 COMMENT:**

Page 16, Discussion: Results should always be interpreted and discussed with caution in mixed-methods study. Because qualitative results can usually not be generalised to the larger population, combining these with quantitative results, which can be generalised, gives an impression that the qualitative results are universally applicable. To avoid this, in the discussion section, qualitative results should be specifically marked as qualitative. For e.g. the authors state that standard of care counseling appeared to fail to reduce alcohol intake, which looks like an universal statement. However, this variable was not analysed in quantitative results and this statement is a result of qualitative data analysis and the same should be stated.

**RESPONSE: Thank you for this comment. In our revised discussion section, we now indicate whether our discussion of the results refers to quantitative or qualitative findings.**

**REVIEWER #2 COMMENT:**

The findings from the manuscript are useful. However, major revision is needed. please refer to the points below:
1. Usually PLWHA is used for “people living with HIV/AIDS”

**RESPONSE:**

**We corrected the abbreviation to “PLWHA” throughout the manuscript as per reviewer’s recommendations.**

**REVIEWER #2 COMMENT:**

2. All the references need to be rewritten and rechecked as there is no uniformity. Moreover, certain references, e.g., 15 are incomplete.

**RESPONSE:**

**We have updated and verified references used in the paper and are now using a uniform style as recommended in the journal.**

**REVIEWER #2 COMMENT:**

3. References need to be re-checked. In the “Introduction”, after 5, immediately 11th reference is coming… the in-between numbers are scattered in the methodology.

**RESPONSE:**

**Thank you for pointing this out. We have updated the references accordingly.**

**REVIEWER #2 COMMENT:**

4. Introduction 2nd paragraph – “Despite its potentially negative impact on the HIV care continuum, unhealthy alcohol use remains largely unaddressed, as evidence-based interventions to reduce alcohol use are rarely implemented in Zambian hospital and clinical facilities (11,12,14) – the studies mentioned are talking about AUD and PLWH; not specifically about Zambia. Thus, the sentence or reference need to be modified accordingly.

**RESPONSE:**

**We agree and have revised this to remove reference to Zambia and insert reference to sub- Saharan Africa. This section now reads (page 2, paragraph 2),”**

**“Despite its negative impact on the HIV care continuum, unhealthy alcohol use remains largely unaddressed in sub-Saharan Africa, as evidence-based interventions to reduce alcohol use are rarely implemented. A systematic review found only 14 studies that used an alcohol-reduction intervention to prevent HIV acquisition in Africa and the results of these studies were mixed. Most interventions in this area have been brief in nature; however, a pilot study in Kenya demonstrated the feasibility of a multi-session alcohol reduction intervention based on cognitive behavioral therapy approaches…”**

**REVIEWER #2 COMMENT:**

5. Quantitative section – what was the need for “second enrolment”? Did the initial ethical approval include drawing of blood samples?

**RESPONSE:**

**Patients only ‘enrolled’ in the parent cohort once;however, the parent cohort informed consent mentioned study visits and sample collection but did not include mention of the qualitative data collection. Hence, when a participant in the parent cohort was invited (a subset of the total) to participate in a qualitative component, we undertook an additional informed consent.**

**In the revised paper we state (page 5, paragraph 2): “Potential participants in the qualitative phase were contacted by a research team member and if agreeable, informed consent (for patients, this was an additional consent on top of the parent cohort consent) was obtained prior to qualitative data collection.”**

**Yes, the initial ethical approval for the parent cohort did include drawing blood samples.**

**REVIEWER #2 COMMENT:**

6. Focus Group Discussions is abbreviated as “FGD’s”. Ethical permission obtained for qualitative analysis?

**RESPONSE:**

**For consistency, we corrected the abbreviation to “FG” throughout the revised manuscript.We obtained ethical permission for qualitative analysis and clarified this in the Ethical Approval section.**

**REVIEWER #2 COMMENT:**

**7.** Methodology section needs to be made more crisp, exclude unnecessary details on how people are counselled at the center etc.

**RESPONSE:**

**We have extensively revised the methodology section as requested (see pages 3-5). We feel it is important that the reader underststand the procedures at the clinic and we have therefore kept limited details on the available counseling, as follows: “Participants received routine ART adherence counseling at each visit. When alcohol was reported, this counseling included brief and unstructured information on alcohol reduction. Patients also received general information on the potential harms of alcohol use during ‘health talks’ delivered by counselors to groups patients waiting in the queue for services.”**

**REVIEWER #2 COMMENT:**

8. The statement before table 1 – “Unhealthy alcohol use was reported at baseline by 280 (40.4%) patients including 26.7% of women and 57.0% of men (P<0.01) and the figures given in table 1 do not match.

**RESPONSE:**

**Thank you. We have fixed the discrepancy. Now we state in the revised results section (page 5, paragraph 4), “*Unhealthy alcohol use was reported at baseline by 280 patients including 36.1 % of women and 63.9% of men (P<0.01).”***

**REVIEWER #2 COMMENT:**

9. In qualitative results, what is WLWH/MLWH/ARVs?

**RESPONSE:**

**We use ARV for antiretroviral and explain this abbreviation at first use. We used “FLWH” for female living with HIV and “MLWH” for males living with HIV. We explain these abbreviations at first use. We also added and “A” to both abbreviations for consistency as per comment # 1 above. Thus, we now use “FLWHA” and “MLWHA” abbreviations throughout the text.**

**REVIEWER #2 COMMENT:**

**10.** What is the difference between clinic A and B – in terms of staff strength, characteristics, treatment or counselling available etc.?

**RESPONSE:**

**They are very similar as both sites have ~5,000 – 10,000 PLWHA in care, both were urban health centers in Lusaka. We unfortunately did not measure staffing levels, competency of staff and also the focus on staff on alcohol as a priority. This would have helped with data interpretation.**

**REVIWER #2 COMMENT:**

11. “UNAIDS 95-95-95” - reference

**RESPONSE:**

**We have updated the references.**

**REVIEWER #2 COMMENT:**

12. Discussion is haphazard and needs to be re-written.

**RESPONSE:**

**We have extensively revised the discussion as requested (see pages 13-14)**
