# Supplementary material for: Alcohol reduction outcomes following brief counseling among adults with HIV in Zambia: A sequential mixed methods study
Source: PLOS Glob Public Health. 2022 May 25;2(5):e0000240. doi: 10.1371/journal.pgph.0000240 (PMC10021288; doi:10.1371/journal.pgph.0000240)
Supplement: Attachment — Submitted filename: Response to Reviewers.docx [file pgph.0000240.s009.docx]

**REBUTTAL LETTER**

**EDITORS COMMENTS**

**EDITOR COMMENT:**

1. Data from sub-Saharan Africa are limited on the impact of alcohol on the HIV epidemic, particularly related to longitudinal responses of people living with HIV (PLWHA) to clinical care that includes counseling on alcohol reduction. The sentence is unclear. Use shorter sentences.

**RESPONSE:**

Data from sub-Saharan Africa on the impact of alcohol on the HIV epidemic in sub-Saharan Africa is limited. In this region, it is not well understood how people with HIV (PLWHA) respond to alcohol reduction counseling while they are linked to HIV clinical care.

**EDITOR COMMENT:**

2. Among 693 participants analyzed. Replace with "Among the data of 693 participants". We do not analyse people but the data.

**RESPONSE:**

The data obtained from 693 participants was analyzed (median age 34 years, 45% men), it revealed that unhealthy alcohol use (AUDIT-C >3 for men; >2 for women) was reported among 280 (40.4%) at baseline and 205 (29.6%) at 1 year on ART.

**EDITOR COMMENT:**

3. Treating unhealthy alcohol integrated with HIV service

**RESPONSE:**

When alcohol use was reported, this counseling included brief and unstructured information on reducing alcohol consumption

**EDITOR COMMENT:**

4. Replace with " We defined unhealthy alcohol use if a participant had an AUDIT-C score of 3-12 for women and 4-12 for men (15), based on World Health Organization (WHO) recommendation"

**RESPONSE:**

The WHO recommends the full AUDIT-C and proposes thresholds (15). We defined moderate alcohol use as 1-2 points for women and 1-3 points for men, while higher points defined unhealthy alcohol use and abstinence was 0 points based on multiple research studies (16).

**REVIEWERS COMMENTS**

**REVIEWER #2 COMMENT:**

**4.** Have the authors made all data underlying the findings in their manuscript fully available (please refer to the Data Availability Statement at the start of the manuscript PDF file)?

**RESPONSE:**

The data are available for use by other researchers but we are unable to upload the data to an open data archive because of Zambian regulatory requirements. If a researcher wants to work with the data, procedures are in place to allow this. First they would contact Dr. Samuel Bosomprah at CIDRZ data center ([Samuel.bosomprah@cidrz.org](mailto:Samuel.bosomprah@cidrz.org)) or the corresponding author. Then, they would be given a data access form to complete. Thereafter, the data can be shared. We can verify that any researcher wouble be provided with the exact data (both quantitative and qualitative) used in the analysis presented in this paper.

**REVIEWER #2 COMMENT:**

**6.** In qualitative section. Utujilijili = tujilijili. References are not yet in one standard format. It would be good if a referencing system, eg., zotero is used. The table for integrating qualitative and quantitative results needs formatting as the results are getting spread across rows.

**RESPONSE:** The term Utujilijili was corrected to tujiljili. The reference section underwent revision via the Mendeley referencing manager. Table 3 was revised clarifying the findings under the various sections.
